# Elucidation of predictors of disease progression in patients with relapsing polychondritis at the onset: potential impact on patient monitoring

**DOI:** 10.1186/s41927-020-00141-8

**Published:** 2020-09-11

**Authors:** Jun Shimizu, Yoshihisa Yamano, Kimito Kawahata, Noboru Suzuki

**Affiliations:** Department of Immunology and Medicine, and Division of Rheumatology and Allergology, Institute of Medical Science, St. Marianna University School of Medicine, Sugao 2-16-1, Miyamae-ku, Kawasaki, 216-8511 Japan

**Keywords:** Auricular involvement, CNS involvement, Relapsing polychondritis, Respiratory involvement

## Abstract

**Background:**

In patients with relapsing polychondritis (RP), organ involvement developed in those with progressive and/or long disease courses. For their management, elucidation of a subgroup suggesting disease progression is awaited.

**Methods:**

We previously conducted a physician’s questionnaire-based retrospective study to elucidate major clinical features of Japanese patients with RP. We here evaluated organ involvement at disease onset and at the last follow-up. We then counted cumulative numbers of involved organs at the last follow-up in 229 RP patients and compared them with involved organ numbers at disease onset, as possible indicators of disease progression. We assigned their prognosis at the last follow-up into “patient prognostic stages” from no medication (stage 1) to death (stage 5). We utilized nonparametric tests for group comparisons.

**Results:**

Involved organ numbers per-patient were 1.13 ± 0.03 at disease onset and 3.25 ± 0.10 at the last follow-up (disease duration was 4.69 ± 0.33 years), and increased along with the patient prognostic stages.

At disease onset, 135 and 48 patients had auricular involvement (59% of 229 patients, defined as auricular-onset subgroup; AO) and respiratory involvement (21%, respiratory-onset subgroup; RO), respectively. 46 patients presented with other conditions (20%, miscellaneous-onset subgroup; MO) including CNS, ocular, and inner ear involvement, among others.

RO patients showed worse (poorer) prognostic stages than AO patients. MO patients developed respiratory and/or auricular involvement thereafter and then showed significantly higher mortality rate (15%; 7/46) than AO patients (5.9%; 8/135).

In RP patients who did not develop respiratory involvement until the last follow-up (throughout the disease course; 117 patients), mortality rate was 19% in 26 MO patients and 3.3% in 91 AO patients. Accordingly, RO patients and MO patients associated with relatively poor prognosis compared with AO patients.

**Conclusions:**

Allocation of patients to RO and MO subgroups was suggested to associate with poorer prognosis of RP than AO subgroups, especially AO subgroups without respiratory involvement throughout. All RP patients deserve careful monitoring but special attention should be paid to MO patients because of their diverse and accelerated disease progression.

## Background

Relapsing polychondritis (RP) is a multisystem disorder characterized by the recurrent inflammation of the cartilage, such as auricular, nasal, respiratory, valvular, and articular chondritis [1]. Other proteoglycan-rich tissues, namely eyes, heart, blood vessels, inner ears, and kidneys were frequently involved [1]. Major causes of death were reported to be respiratory complications, such as pneumonia and airway collapse [2, 3]. A recent clustering analysis described that RP patients with respiratory involvement demonstrated relatively lower mortality rate than the previous studies probably because of improved management [4]. Various symptoms with several clinical clusters make it difficult to diagnose and studies now focus on the potential room of imaging technique for the diagnosis of RP [5], as well as for evaluating activity and extent of the disease.

We conducted an epidemiological survey of 239 Japanese RP patients in 2009 and collected clinical information [6]. We found a strong inverse relationship between the incidence of respiratory involvement and that of auricular involvement, suggesting that patients with respiratory involvement and patients with auricular involvement were mutually exclusive [7].

We then divided the patients into 3 subgroups, namely patients with respiratory involvement (without auricular involvement), patients with auricular involvement (without respiratory involvement), and patients with both respiratory and auricular involvement at the last follow-up [8]. Patients with both respiratory and auricular involvement suffered frequently from progressive and long disease courses. Indeed, some patients with mild diseases at the onset developed more aggressive diseases having both respiratory and auricular involvement. Predictors of disease progression are awaited for establishing better clinical management.

Here, we compared severity of RP at disease onset with that at the last follow-up to elucidate disease progression including possible termination due to death. We investigated whether one of the most significantly associated factors at disease onset with overall mortality rate served as a predictor of disease progression.

## Methods

### A multi-institutional study survey with a subgroup analysis

We conducted a retrospective cohort study using a physician questionnaire in Japanese major medical facilities to characterize the epidemiology, clinical features, and outcome of RP [6]. All physicians who got the questionnaire were informed the purpose of the study and their questionnaire responses would be kept confidential [6]. Onset of RP was assessed according to McAdams criteria [2]. The physicians performed tissue biopsies in 95.4% patients. 60.5% of the patients who underwent biopsy were histologically diagnosed as having RP [6]. 52.1% of the patients were screened for the inflammation with computed tomography, magnetic resonance imaging, and/or scintigraphy [6].

The questionnaire consisted of 5 sections, namely patients’ profiles, clinical features, laboratory findings, treatment, and prognosis. In the clinical feature section, physicians examined the involved organs at disease onset and at the last follow-up by assigning into 10 items, namely 1) auricular, 2) nasal, 3) inner ear, 4) joint, 5) ocular, 6) respiratory, 7) skin, 8) cardiovascular, 9) central nervous system (CNS), and 10) renal involvement. In this study, we counted cumulative numbers of involved organs, and then calculated their average (on a per-patient basis) in each subgroup. We considered them as a possible indicator of disease progression for comparison.

The prognosis was assigned into 5 items, namely 1) no medication, 2) well-controlled, 3) limited responses, 4) progressive disease courses, and 5) death. We considered such a prognostic stage value as a quantitative indicator of disease severity.

We previously found that auricular involvement and respiratory involvement were two major independent clinical features in the assessment of disease progression [7, 8]. To characterize disease progression of RP in the current study, we evaluated the parameters (mean numbers of involved organs on a per-patient basis, prognosis stage values, and mortality rates) based on the “space”, namely auricular involvement and respiratory involvement, and the “time”, namely the disease onset and the last follow-up [9].

### Statistical analysis

We utilize several nonparametric tests in this study [10–12]. We compared age, disease duration, involved organ numbers, and prognostic stages using Steel-Dwass test. We compared the incidence of organ involvement using dummy variables. The values 0 and 1 indicated the absence and presence of organ involvement, respectively, and were compared by Steel-Dwass test. We compared mortality rates using chi-square and Fisher’s exact tests. These parameter titers were expressed as mean ± standard error of the mean. *P* value less than or equal to 0.05 was considered significant. We used software JMP 13.0.0 (SAS Institute Japan, Tokyo, Japan) for statistical analysis.

## Results

We conducted an epidemiological survey and obtained data of 239 RP patients in 2009 [6]. In the current study, we excluded 6 patients from the recruited 239 patients because of lack of relevant data. After preliminary analysis, we decided to exclude additional 4 patients with both auricular and respiratory involvement at disease onset to simplify the analysis. Thus, we conducted this study with total 229 patient data.

One of 229 patients was positive for myeloperoxidase antineutrophil cytoplasmic antibody with laryngeal chondritis and had no signs of systemic vasculitis. Due to the lack of symptoms of vasculitis, we included the patient in this study.

### Calculation of involved organ numbers on a per-patient basis at disease onset and at the last follow-up

**Fig. 1:**
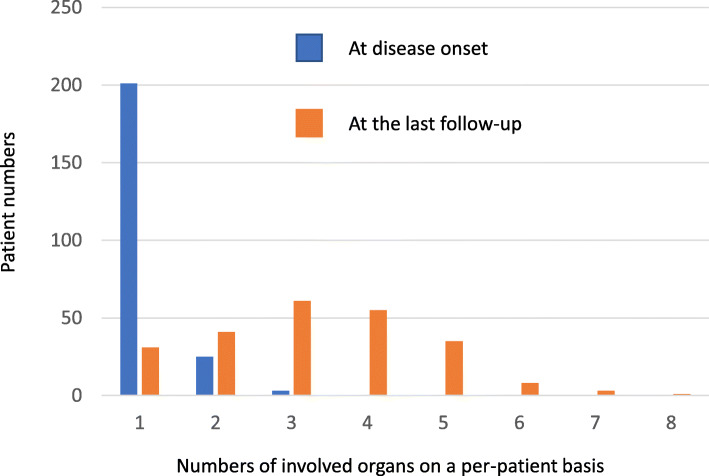
Involved organ numbers per-patient at disease onset and at the last follow-up in 229 patients with RP. The involved organ numbers per-patient was 1.13 ± 0.03 at disease onset. At the onset, 201 patients had single manifestation and 28 patients had multiple manifestations. Cumulative numbers of involved organs at the last follow-up were 3.25 ± 0.10. Disease duration from the first symptom to the last follow-up was 4.69 ± 0.33 years

We counted involved organ numbers of each patient and calculated their averages in 229 RP patients (Fig. 1). Involved organ numbers were 1.13 ± 0.03 organs at disease onset. At the onset, 201 patients had single manifestation and 28 patients had multiple manifestations. Cumulative numbers of involved organs were 3.25 ± 0.10 organs at the last follow-up. Disease duration from the first symptom to the last follow-up was 4.69 ± 0.33 years. These findings support that cumulative numbers of involved organs per-patient may be a possible indicator of disease progression.

### Significant association of cumulative numbers of involved organs per-patient with their prognostic stages

Physicians were asked to determine prognostic stages from 5 items of prognosis in the section of the questionnaire, namely 1) no medication (11 patients, 4.8% of 229 patients), 2) well-controlled (155 patients, 68%), 3) limited responses (32 patients, 14%), 4) progressive disease courses (9 patients, 3.9%), and 5) death (22 patients, 9.6%).

**Fig. 2:**
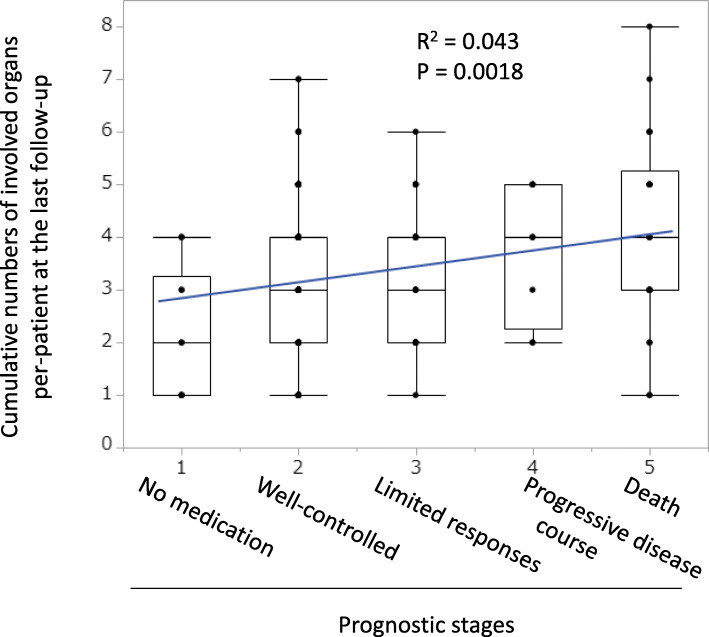
Comparison of cumulative numbers of involved organs per-patient and their prognostic stages. Patient clinical prognosis was assigned into 5 stages, namely 1) no medication, 2) well-controlled, 3) limited responses, 4) progressive disease courses, and 5) death. We assessed the relationship between cumulative numbers of involved organs per-patient and their prognostic stages. We found that the numbers of involved organs per-patient increased along with the prognosis stages. A box plot of each subgroup of RP patients and a regression line were indicated

We assessed the relationship between cumulative numbers of involved organs per-patient and their prognostic stages. The prognostic stages positively correlated with cumulative numbers of involved organs per-patient (Fig. 2). We found that the numbers of involved organs increased corresponding to the prognosis stages.

### Evaluation of involved organs at disease onset and at the last follow-up

We then evaluated organ involvement at disease onset and at the last follow-up in detail.

At the onset, 135 patients had auricular involvement (59% of 229 patients, auricular-onset subgroup; abbreviated as AO) and 48 patients had respiratory involvement (21%, respiratory-onset; RO). 46 patients presented with other conditions (20%, miscellaneous-onset; MO), namely ocular involvement (18 patients, 7.9%), inner ear dysfunction (9 patients, 3.9%), joint involvement (8 patients, 3.5%), CNS involvement (6 patients, 2.6%), nasal chondritis (3 patients, 1.3%), and skin involvement (2 patients, 0.87%). We found no significant differences in gender, age, age at disease onset, and disease duration among AO, RO, and MO subgroups (Table 1). Excluded 4 patients from this study with auricular and respiratory involvement at disease onset had moderate clinical courses (mean age at onset, 58.0 ± 15.9 years; disease duration, 6.25 ± 1.25 years; prognostic stages, 2.00 ± 0.00; cumulative numbers of involved organs, 3.33 ± 0.28 organs).

**Table 1:** Patient demographic data of AO^a^ (Auricular-Onset) subgroup, RO^a^ (Respiratory-Onset) subgroup, and MO^a^ (Miscellaneous-Onset) subgroup

	AO group	RO group	MO group
Patient number (n)	135	48	46
Gender (male-female)	67:68	24:24	28:18
Mean age	56 ± 1.5^b^	57 ± 2.2	60 ± 2.2
Mean age at onset	52 ± 1.5	52 ± 2.2	56 ± 2.3
Disease duration, years	4.9 ± 0.5	5.3 ± 0.7	3.6 ± 0.4

^a^AO: patients with auricular involvement at disease onset (Auricular-Onset), RO: patients with respiratory involvement at disease onset (Respiratory-Onset), MO: patients with miscellaneous organ involvement at disease onset (Miscellaneous-Onset). 4 patients with both respiratory and auricular involvement at disease onset were excluded from this study due to their small numbers, to simplify the analysis. ^b^Age, age at onset, and disease duration were expressed as mean ± standard error of the mean

We found no significant differences in the use of immunosuppressants (prednisolone, methotrexate, cyclophosphamide, cyclosporine A, and azathioprine) and biologics (etanercept, adalimumab, and tocilizumab) among the 3 subgroups, except for infliximab. Infliximab was frequently administered in RO patients (11.8 ± 0.05%) compared with AO patients (2.18 ± 0.01%) and MO subgroup (0.00 ± 0.00%).

At the last follow-up, 117, 47, and 65 patients had auricular involvement (51% of 229 patients, auricular-the last subgroup; AL), respiratory involvement (21%, respiratory-the last subgroup; RL), and both respiratory and auricular involvement (28%, both-the last subgroup; BL), respectively.

Patients involved in this study demonstrated either respiratory involvement, auricular involvement, or both involvement at the last follow-up without exception. 199 patients (87%) had extra-respiratory and extra-auricular conditions at the last follow-up as we previously reported [6, 13–15].

We evaluated the incidence of extra-auricular and extra-respiratory manifestations at the last follow-up based on the subgroup analysis at the onset (Table 2). The incidence of ocular and inner ear involvement were significantly higher in MO patients than those in AO and RO patients (Table 2). The incidence of CNS and cardiovascular involvement were significantly higher in MO patients than those in RO patients (Table 2).

**Table 2:** Clinical manifestations at the last follow-up in AO, RO, and MO subgroups

	Auricular involvement, n, (% of the group)^a^	Respiratory involvement, n, (%)^b^	Ocular involvement, n, (%)^c^	Nasal involvement, n, (%)	Joint involvement, n, (%)	Inner ear involvement, n, (%)^c^	Skin involvement, n, (%)	CNS involvement, n, (%)^d^	Renal involvement, n, (%)	Cardiovascular involvement, n, (%)^d^
All 229 patients	182, (79%)	112, (49%)	113, (49%)	91, (40%)	90, (39%)	64, (30%)	32, (14%)	28, (12%)	18, (7.9%)	16, (7.0%)
AO, 135 patients	135, (100%)	44, (33%)	60, (44%)	49, (36%)	60, (44%)	32, (24%)	19, (14%)	17, (13%)	10, (7.4%)	10, (7.4%)
RO, 48 patients	9, (19%)	48, (100%)	17, (35%)	25, (52%)	13, (27%)	8, (17%)	5, (10%)	1, (2.1%)	2, (4.2%)	0, (0.0%)
MO, 46 patients	38, (83%)	20, (43%)	34, (74%)	15, (33%)	16, (35%)	24, (52%)	8, (17%)	10, (21%)	6, (13%)	6, (13%)

^a^The incidence was significantly higher in AO patients than that in RO patients and that in MO patients. The incidence was significantly higher in MO patients than that in RO patients

^b^The incidence was significantly higher in RO patients than that in AO patients and that in MO patients

^c^The incidence was significantly higher in MO patients than that in AO patients and that in RO patients

^d^The incidence was significantly higher in MO patients than that in RO patients

27 of 28 RP patients with CNS involvement (96% of 28 patients) were derived from AO and MO patients and, at the last follow-up, 25 (89%) and 3 (11%) patients were allocated to AL and BL subgroups, respectively. No RP patients with CNS involvement fell into RL subgroup. In the MO subgroup, 6 patients presented with meningitis/encephalitis preceding any detectable chondritis and developed auricular chondritis later in the disease course.

### Comparison of mortality rates among 3 patient subgroups at disease onset

Mortality rate was obtained in each of 3 patient subgroups at disease onset. We then calculated the rate in each of 3 patient subgroups at the last follow-up (Table 3).

**Table 3:** Involved organs at disease onset and at the last follow-up, and the mortality rates

Onset	Mortality rates (Death)	Last follow-up	Mortality rates (Death)
AO, 135 patients	5.9% (8)^a^	AL^b^, 91 patients	3.3% (3)
		BL^b^, 44 patients	11% (5)
RO, 48 patients	15% (7)	RL, 39 patients	13% (5)
		BL^b^, 9 patients	22% (2)
MO, 46 patients	15% (7)^a^	AL, 26 patients	19% (5)
		RL, 8 patients	0% (0)
		BL, 12 patients	17% (2)

^a^Mortality rate was significantly higher in MO patients than that in AO patients (P = 0.048). ^b^AL: patients with auricular without respiratory involvement at the last follow-up (Auricular-Last), RL: patients with respiratory without auricular involvement at the last follow-up (Respiratory-Last), BL: patients with both respiratory and auricular involvement at the last follow-up (Both-Last). MO patients demonstrated poor prognosis even without respiratory involvement, namely 26 MO-AL patients, at the last follow-up

The mortality rate was 5.9% in 135 AO patients. In the AO patients, 91 patients (67% of 135 patients) and 44 (33%) were allocated into BL and AL subgroups, with 3.3% (3/91) and 11% (5/44) mortality rates, respectively.

The mortality rate was 15% in 48 RO patients. In the RO patients, 39 patients (81% of 48 patients) and 9 (19%) were allocated into RL and BL subgroups, with 13 and 22% mortality rates, respectively.

46 MO patients showed a high mortality rate (15%, 7/46) and the mortality rate was significantly higher than AO patients (*P* = 0.048) (Table 3). Their causes of death included encephalitis, myocardial infarction, and a chronic myeloproliferative disorder

We then focused on RP patients without respiratory involvement throughout their courses. To this end, patients of MO and AO subgroups who did not have respiratory involvement until the last follow-up (117 patients) were analyzed. Mortality rate was significantly higher in MO patients (19%) than that in AO patients (3.3%, *P* = 0.013) (Table 3).

We recognized 7 patients with hematologic malignancies and 5 patients with solid malignancies in the 229 patients. They consisted of 6 AO, 3 RO, and 3 MO patients and their mortality rate was 25% (3/12).

### Comparison of cumulative numbers of involved organs among the 3 patient subgroups at the onset

**Fig. 3:**
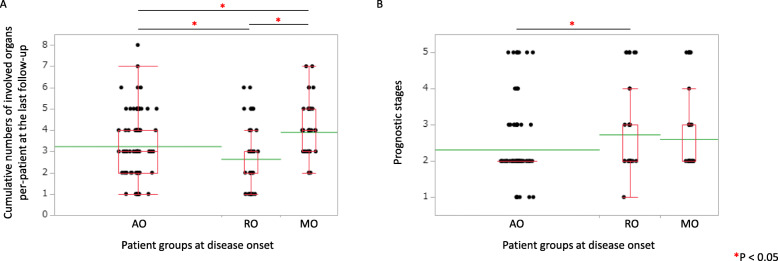
Comparison of involved organ numbers and prognostic stages among 3 patient subgroups at disease onset. We compared cumulative numbers of involved organs per-patient at the last follow-up (**a**) and prognostic stages at the last follow-up (**b**) among 3 patient subgroups initially assigned at disease onset; AO, RO, and MO subgroups. **a** When we compared cumulative numbers of involved organs per-patient at the last follow-up among AO, RO, and MO patients, the numbers were significantly higher in MO patients than those in AO patients and RO patients. The numbers were significantly higher in AO patients than those in RO patients. We found that the MO patients were followed thereafter by respiratory involvement and/or auricular involvement without exception (Table 3).**b** When we compared prognostic stages at the last follow-up among AO, RO, and MO patients, the stage values were significantly larger in RO patients than those in AO patients. A box plot (red line) and a mean level (green line) of each subgroup of RP patients were indicated

When we compared cumulative numbers of involved organs per-patient among AO, RO, and MO patients, the numbers were significantly higher in MO patients (3.91 ± 0.18) than those in AO patients (3.24 ± 0.13, *P* = 0.032) and RO patients (2.65 ± 0.21, *P* < 0.001) (Fig. 3a). The numbers were significantly higher in AO patients than those in RO patients (*P* = 0.030). We found that the MO patients were followed thereafter by respiratory involvement and/or auricular involvement without exception (Table 3).

In the analysis of MO and AO patients who did not have respiratory involvement until the last follow-up, MO patients had significantly higher cumulative numbers of involved organs per-patient (3.88 ± 0.17) than AO patients (2.92 ± 0.15, *P* = 0.012).

### Comparison of prognostic stages among the 3 patient subgroups at the onset

When we compared prognostic stages at the last follow-up among AO, RO, and MO patients, the stage values were significantly larger in RO patients (2.73 ± 0.16) than those in AO patients (2.32 ± 0.08, *P* = 0.035) (Fig. 3b), suggesting poorer prognosis of RO patients.

In the analysis of MO and AO patients who did not have respiratory involvement until the last follow-up, MO patients showed significantly worse (poorer) prognostic stages (2.73 ± 0.23) than AO patients (2.09 ± 0.07, *P* = 0.029).

Collectively, MO patients (even without respiratory involvement throughout) and RO patients showed relatively poorer prognosis compared with AO patients.

## Discussion

RP is a remittent and episodic disease and extent of the tissue damage increases with time [2–4, 6, 16]. Mortality rates were reported to increase in patients with respiratory involvement [2–4, 6], cardiovascular involvement [2–4, 15], CNS involvement [2, 13], and hematological disorders [4]. We found that cumulative numbers of involved organs at the last follow-up associated positively with prognostic stages (Fig. 2). In this study, we suggest that the cumulative numbers of involved organs at the last follow-up, as well as prognostic stages, may be a predictor of disease progression in RP.

We found that the overall prognosis of RO patients was poorer than that of AO patients (Fig. 3b). Recently, it has been proposed that patients with respiratory involvement were distinctive from other RP patients in view of their clinical characteristics, responses to the treatment, and the prognosis [17]. Our current finding supports this proposal.

We observed that the cumulative numbers of involved organs at the last follow-up were higher in MO patients than those in AO patients and RO patients (Fig. 3a). Actually, 46 MO patients were allocated into AL (Auricular-Last) subgroup (54%, 25 of 46 patients), RL (Respiratory-Last) subgroup (17%, 8 patients), and BL (Both-Last) subgroup (28%, 13 patients) (Table 3). The incidence of ocular and inner ear involvement were significantly higher in MO patients than that in AO and RO patients (Table 2). The incidence of CNS and cardiovascular involvement were significantly higher in MO patients than that in RO patients (Table 2).

The MO patients showed a significantly higher mortality rate (15%) than AO patients (5.9%) (Table 3). Previous studies demonstrated that miscellaneous-onset RP patients were identified with CNS involvement [18] and skin involvement [19, 20] at the onset, both of which were suggested to associate with severe complications [18–20]. Miscellaneous disorders without chondritis may be challenging to diagnose in patients with RP. Absence of ear and nasal involvement was reported to lead to the diagnostic delay [21].

We found that 12% (28 patients) of 229 patients with RP developed CNS involvement. 6 patients (21% of 28 patients) with CNS involvement at disease onset presented with meningitis/encephalitis preceding any detectable chondritis and developed auricular chondritis later in the disease course. In the 28 patients, 25 (89%) and 3 (11%) patients with CNS involvement were allocated to AL and BL subgroups at the last follow-up, respectively, and no patients fell into RL subgroup. Again, CNS involvement and respiratory involvement looked mutually exclusive [7].

It was reported that CNS involvement occurred in 8–12% of RP patients [2, 4, 13, 16] and the mortality rates were high [13, 18, 22, 23]. When we reviewed the literature [18, 22–42], 14 of 50 RP patients (28%) with meningitis/encephalitis developed neurological symptoms preceding any detectable chondritis (Table 4). At the last follow-up, 82 and 18% of the patients with CNS involvement were allocated to AL and BL subgroups, respectively (Table 4), consistent with our finding of close relationship between auricular involvement and CNS involvement [8].

**Table 4:** Relationships between meningitis/encephalitis and chondritis in RP patients based on a review of the literature^a^

	Mean age at onset (y)	Male to female ratio	Time interval^**b**^ (months)	Follow-up^**c**^ (months)	Onset			Last follow-up		
					AO	RO	MO	AL	RL	BL
All patients (*n* = 50)	56 ± 1.5^d^	3.2:1	NA^e^	13 ± 2.0	34	4	22	41	0	9
Patients presented with meningitis/encephalitis and developed chondritis later (*n* = 14)	57 ± 3.1	3.7:1	6.2 ± 1.6	8.5 ± 2.1	0	0	3	13	0	1
Patients presented with chondritis and developed meningitis/encephalitis later (*n* = 17)	58 ± 2.3	7.5:1	12 ± 4.3	18 ± 4.3	16	1	7	14	0	3
Patients presented with both meningitis/encephalitis and chondritis (*n* = 19)	55 ± 2.4	1.7:1	NA	11 ± 3.5	18	3	12	14	0	5

^a^A review of the literature was conducted on RP meningitis/encephalitis using PubMed [18, 22–42]

^b^Time interval (months) between the onset of meningitis/encephalitis and that of chondritis

^c^Follow-up duration of patients (months)

^d^Age, time interval, and follow-up duration were expressed as mean ± standard error of the mean

^e^NA, Not applicable

Our findings support the notion that RP patients with respiratory involvement relate to poorer prognosis [2, 3, 6, 17]. It has been generally accepted that early detection of and treatment on respiratory involvement are important to reducing mortality rate of RP [17].

This study has a limitation because of its retrospective study design. We obtained the clinical data at disease onset and at the last follow-up. Further studies are needed to elucidate the pathological consequence of inflammation of patients with RP, especially focusing on the insidious disease progression.

## Conclusions

When we analyzed clinical information of 229 RP patients with a focus on the disease onset, mortality rates were worse in RO (Respiratory-Onset) patients and MO (Miscellaneous-Onset) patients than AO (Auricular-Onset) patients. When we analyzed the data in 117 patients without respiratory involvement throughout their disease course, MO patients showed poorer prognosis than AO patients. These findings suggest that MO patients were considered to be exposed to high-risk of disease progression with poorer prognosis.

## Data Availability

The datasets used and/or analyzed during the current study are available from the corresponding author on reasonable request.

## Abbreviations

AL: Patients with auricular involvement at the last follow-up
AO: Patients with auricular involvement at disease onset
BL: Patients with both respiratory and auricular involvement at the last follow-up
CNS: Central nervous system
MO: Patients with miscellaneous organ involvement at disease onset
RL: Patients with respiratory involvement at the last follow-up
RO: Patients with respiratory involvement at disease onset
RP: Relapsing polychondritis
